# SLOfit Lifelong: A model for leveraging citizen science to promote and maintain physical fitness and physical literacy across the lifespan

**DOI:** 10.3389/fpubh.2022.1002239

**Published:** 2022-09-06

**Authors:** Gregor Jurak, Shawnda A. Morrison, Maroje Soric, Bojan Leskošek, Marjeta Kovač, Tjaša Ocvirk, Vedrana Sember, Jaka Kramaršič, Kaja Meh, Žan Luca Potočnik, Rok Blagus, Neja Markelj, Petra Golja, Vojko Strojnik, Vedran Hadžić, Gregor Starc

**Affiliations:** ^1^Faculty of Sport, University of Ljubljana, Ljubljana, Slovenia; ^2^Faculty of Kinesiology, University of Zagreb, Zagreb, Croatia; ^3^Institute for Biostatistics and Medical Informatics, Faculty of Medicine, University of Ljubljana, Ljubljana, Slovenia; ^4^Faculty of Mathematics, Natural Sciences and Information Technologies, University of Primorska, Koper, Slovenia; ^5^Biotechnical Faculty, University of Ljubljana, Ljubljana, Slovenia

**Keywords:** aging, exercise monitoring, participatory research, policy making, population health, surveillance

## Abstract

SLOfit Lifelong is a public health initiative which was created to upgrade a well-established, national physical fitness surveillance system for Slovenian schoolchildren that has been collecting annual fitness and health data for over three decades. The ultimate objective of creating SLOfit Lifelong was to build a modern societal infrastructure with the capacity and ability to detect future causal associations between childhood physical fitness trends and future health outcomes based on the lifelong surveillance of one's own fitness status. By instilling citizens with an ambition to test, understand, and follow-up their own physical fitness and health status (including related health risk factors), this initiative provides the technical support and expert feedback needed to engender greater individual control over understanding (and thus modulating), one's own physical fitness status as they progress into older adulthood. This perspective paper details the extensive approach taken to devise appropriate fitness test batteries for adults and older adults which can also relate to the student version of the original SLOfit test database, including establishing criterion health risk zones and a public approach to establish this national, citizen-driven health feedback framework. Through its sophisticated online web applications, social media, print media, and outreach workshops, SLOfit Lifelong provides the expert support for public health engagement by fostering positive lifelong physical literacy experiences an individual can enjoy across their aging journey.

## Introduction

Cardiovascular diseases (CVD) have become the leading cause of global mortality in the 21st century primarily due to lifestyle changes, including increased physical inactivity (1, 2). Recent estimates find the direct health care costs of physical inactivity in Europe reach upwards of 11.7 billion euros per year, with an additional 3.8 billion being lost to poor productivity (3). Although CVD events occur more frequently during, or after, the fifth decade of life, there is strong evidence that the precursors of CVD originate in childhood and adolescence (4, 5) and can be tracked into adulthood (6). Amongst the most important modifiable CVD risk factors is physical fitness. Being physically fit means being able to carry out daily tasks with vigor and alertness (7). Physical fitness refers to a full range of physical qualities, including cardio-respiratory fitness, muscular strength, speed of movement, agility, coordination, flexibility, and body composition. Having higher childhood fitness provides lower risk of developing CVD later in life and lower risk of premature death (8). Increased physical fitness in childhood is associated with positive CVD risk factors (9), improved mental health (10), and quality of life (11, 12).

With aging, negative physiological and physical changes can occur, including sarcopenia (13), progressive muscle mass loss (14), and declines in muscular strength (15, 16), cardiovascular function (17), flexibility (18), and balance (19). These can cause functional declines that prevent older people from performing basic tasks (20–22), affecting mortality rates (23), increasing fall risk, and leading to higher incidence of hospitalization (24). Physical fitness is clearly an important health marker across the lifespan (25). The American Heart Association recommends checking fitness as part of general check-ups (26). Therefore, monitoring physical fitness across the life course is critical not only to prevent chronic disease, but also to maintain functional capacity, identify weaknesses, and addressing them early enough to possibly reverse declines *via* lifestyle interventions.

The purpose of this paper is to communicate how SLOfit Lifelong was developed, the health data it conveys, and how this initiative collaborates with individuals to provide quality testing and expert feedback for adults of all ages who wish to better understand their body and its functional capacity across their lifetime.

## Ethics, setting context and population

### Ethics statement

Any data collected within the context of SLOfit Lifelong was approved by the Slovenian National Medical Ethics Committee (ID: 0120-468/2021/3), following the Declaration of Helsinki. Participants provided written, informed consent prior to data collection.

### Setting context and population

The original SLOfit infrastructure program (herein differentiated as: SLOfit Students) monitors child fitness through their entire schooling period (27). This includes roughly *N* = 13,000 graduates per year who are potential SLOfit Lifelong cohort members. SLOfit Lifelong is designed to be capable of housing their fitness data from childhood to adulthood, thus “slotting” in existing SLOfit members as they age, in parallel to recruiting new participants.

In Slovenia, the physical fitness of children and adolescents is monitored by a program originally entitled “Sports Educational Card”, implemented in 1982 on a sample of Slovenian schools. After 5 years of refinement and pilot testing, the program was introduced to all Slovenian primary and secondary school curricula in all Slovenian schools (28). “SLOfit Students” is one of the oldest and largest child and youth fitness databases in the world, holding information on more than 40 birth cohorts who have been followed for ~13 years. Exact measurement protocols are published in the Sports Educational Card manual (29), a compulsory tool for every PE teacher freely available on the SLOfit website (www.slofit.org/ucitelj/administracija). Testing is administered by PE teachers trained from the Faculty of Sport, University of Ljubljana, which is the only institution in Slovenia educating PE teachers in a 5-year specialist program. All schools follow identical testing protocols and use standardized equipment. Results are entered into the “My SLOfit app” where data is first checked with smart algorithms, analyzed, and made available to all registered users, i.e., parents/guardians control access (doctors, coaches, the children themselves).

Beginning in 2016, SLOfit researchers developed and launched a free-for-use, online application system called “My SLOfit” (27). Amongst other features, this application allows users to store their personal data long after their schooling period has ended. The My SLOfit app supports 24-h movement behavior data collection and lifestyle habits (e.g., physical activity, sleep, sedentary time), in addition to direct fitness testing data. SLOfit Lifelong now has the potential to house novel data on young people graduating secondary education (18–19 y) and continue as long as they wish.

## Key programmatic elements

### Medical screening

We developed a medical screening protocol to mitigate health-risks associated with fitness testing for adults with specific/underlying clinical concerns. SLOfit Lifelong test batteries include preliminary medical screenings performed at rest, before exercise, for: (1) arterial blood pressure (2) blood oxygen saturation (3) heart rate, (4) medical history questionnaire (5) short health screening questionnaire. Adults visit a center of their choice (e.g., local gymnasium or school) and a fitness tester administers the assessment. The “head of testing” is an exercise expert with (at minimum) a bachelor's degree in kinesiology, physical education, physical training, physiotherapy, or medicine. Health screening questionnaire is an adapted PAR-Q form [American College of Sports Medicine (30)]. Next, participants complete anthropometry measurements, including a brief consultation with the head of fitness testing on their fitness status and potential health risks. Generally, adults between 19 and 64 y who are deemed low-risk can complete SLOfit Adult testing onsite.

Participants completed a warm-up to a prepared video based on age and fitness level of the individual. After warm-up, participants begin the motor tasks. Although testing order is not strictly specified in SLOfit Students or SLOfit Seniors (except that the cardiorespiratory endurance test should be performed last), for SLOfit Adults tests do follow a specific order, namely: vertical jump (leg power), Figure-8 run (agility), handgrip (arm strength), arm plate tapping (coordination), sit and reach (flexibility), partial curl-up (core strength), and a 6-min walk (cardio-respiratory endurance).

### Fitness tests battery identification and selection process

A working group was assembled (*N* = 14–16 senior and junior researchers) at Faculty of Sport, Ljubljana to identify, pilot test and define appropriate fitness tests for adults. Discussions were then held with experts from target end-users, e.g., fitness industry, sports clubs, national public health institute, patients, academia, and educators familiar with SLOfit. The entire test battery development occurred over ~36 months, divided into 5 steps. First, the work group reached consensus on which fitness components needed monitoring. Seven components were prioritized: body composition/adiposity, cardiorespiratory endurance, muscular strength, power, coordination, agility, and flexibility. Second, it was decided to split adult test batteries to encompass advancing age and variable fitness levels. Three distinct batteries were decided for: (a) Young Adults (19–34 y), Adults (35–64 y), and Seniors (65+ y). The group then cross-referenced whether any existing published test batteries fit the specific needs of SLOfit Lifelong and found that none matched the existing SLOfit Student version well enough in terms of breadth or scope of fitness tests included. Thus, step 4 was finding the range of appropriate tests and conducting pilot measurements to assess testing feasibility (e.g., measurement duration, positive participant feedback). In the final step 5, three fitness testing batteries were defined: (a) *SLOfit Young Adults* (19–34 y) (29); (b) *SLOfit Adults* (35–64 y) and (c) *SLOfit Senior* (65+ y) (31). When identifying optimal fitness tests for adults, we searched for test with acceptable reliability and validity, while maintaining feasibility inherent to large scale filed testing. A key prerequisite was also that the components of fitness measured by the adult program should align with SLOfit Students so future comparisons could be extrapolated.

### Pilot testing

SLOfit Lifelong testing was piloted on each adult group to confirm whether the selected tests were appropriate and determine the best test order for successfully completing all sections with positive vigor. All in-person testing took place at the Faculty of Sport or Faculty of Education, University of Ljubljana, Slovenia, following national guidelines for COVID-19 safety protection.

The test batteries were first piloted on university students (26.04.2021–12.05.2021). Next, Young Adults (*N* = 471) and Adults (*N* = 527) were sampled between May 2021 and June 2022, including ~20 measurement days at the University. Seniors (*N* = 152) were pilot tested last, from 31.03.2022 to 21.06.2022. On 21.04.2022, measurements were performed at the Coronary Club of Ljubljana with participants having diagnosed cardiovascular disease (s). After this first stage, some tests were modified or improved slightly. Due to problems with feasibility (e.g., long duration and difficulties with standardization of the protocol), with the Sorenson test and *T*-test prompted replacement with “partial curl-up” and “Figure 8 run”, respectively (Table 1). Statistical or other pilot test details are available upon request to the corresponding author.

**Table 1:** Fitness test battery components for each age group included in the SLOfit Lifelong community health program.

		**SLOfit young adults (18–34 y)**	**SLOfit adults (35–64 y)**	**SLOfit seniors (65+ y)**
**Fitness test component**	**Test**			
Health screening tests	- Arterial blood pressure at rest	x	x	x
	- Oxygen saturation	x	x	x
	- Resting heart rate	x	x	x
	- Health screening questionnaire	x	x	x
	- Health history questionnaire	x	x	x
Anthropometry	- Body height	x	x	x
	- Body weight	x	x	x
	- Waist circumference	x	x	
Health-related physical fitness measures	−600-m run	x		
	−6-min walk test		x	x
	−2-min step test			x
	- Sit-ups	x		
	- Stand and reach	x		
	- Chair sit and reach test			x
	- Back scratch test			x
	- Bent arm-hang	x		
	- Hand grip test	x		
	- Partial curl-up test		x	
	- Sit and reach		x	
	−30-s chair stand			x
Skill-related physical fitness	−20-s arm plate tapping	x	x	
	- Standing long jump	x		
	- Polygon backwards obstacle course	x		
	−60-m run	x		
	- Standing vertical jump		x	
	−30-s arm curl			x
	- Timed “up and go” test			x
	- Figure 8 run		x	

All adults between the ages of 19 and 35 can complete the Sports Educational Chart (i.e., SLOfit Student protocol) or older adults can choose one of the SLOfit Adult tests if the head tester determines that the participant is low risk and free of any major health issues. Participants completing the Sports Educational Chart can thus directly compare their results to those completed during their schooling period. At any point a specific test can be omitted or added to each fitness battery upon request. Alternatively, participants can complete a SLOfit Adults protocol and two fitness tests from Sports Educational Chart if they desire, to compare their results from primary or secondary school for a specific test.

### Determination of criterion references—healthy fitness zones

We performed literature searches for studies published (from 1.1.1980) that investigated the ability of a specific test to predict mortality or morbidity (i.e., metabolic, cardiovascular, and bone disease or cancer) to identify cut-off values related to health risk for each test included. The search was built around two areas: (1) the specific test and (2) health-related outcomes. There were criterion-referenced standards for BMI, waist circumference, and handgrip strength. The standards for BMI and waist circumference did not vary across age groups, but handgrip strength did differ for those >65 y compared to prior age groups.

**Figure 1:**
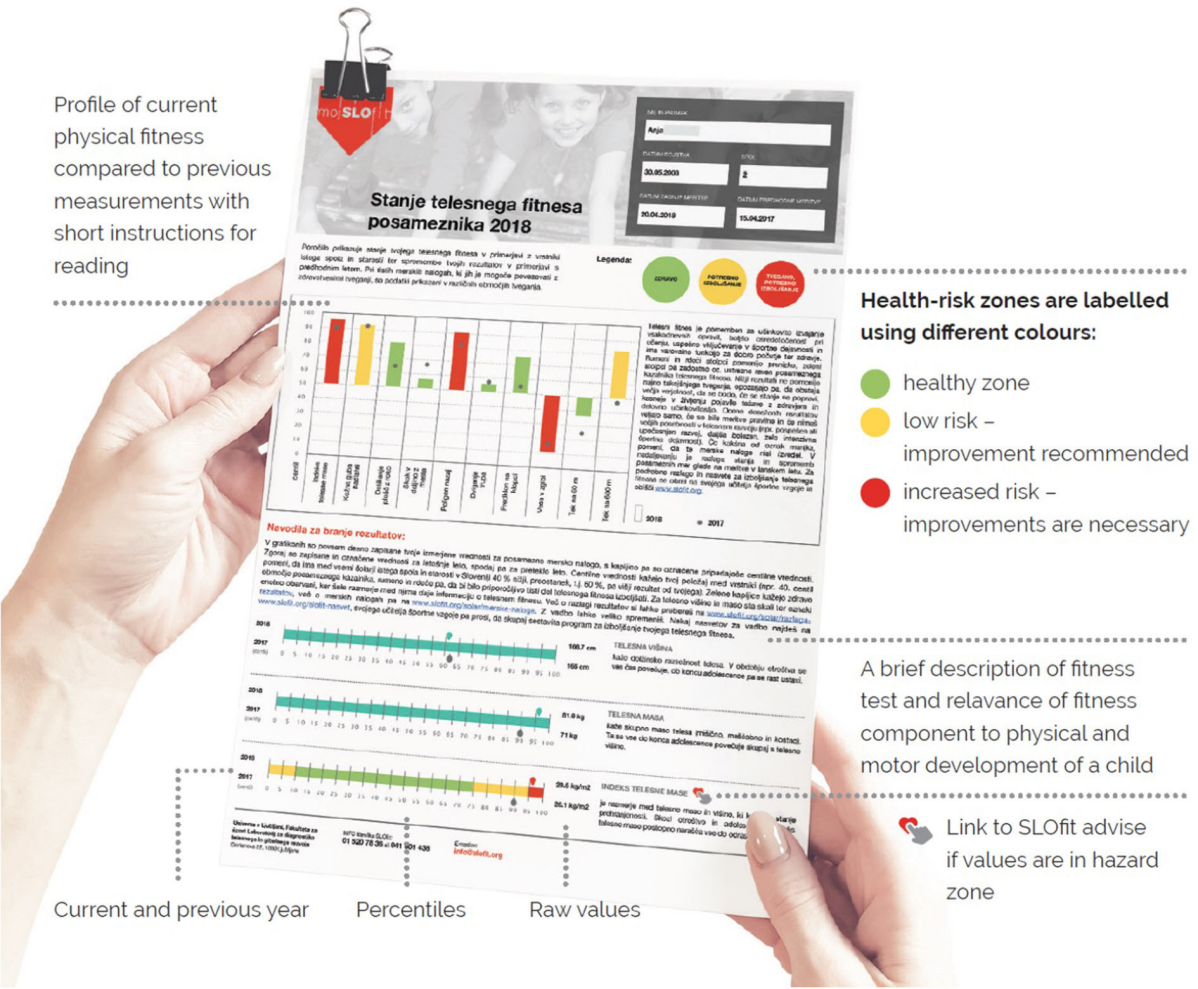
SLOfit Lifelong health risk feedback report example. The free-of-charge My SLOfit app provides all registered users with official SLOfit reports that specify healthy fitness outcomes, centiles, and participant histories. SLOfit reports help facilitate better and more concise communication between the individual and other important stakeholders, e.g., family, teachers, medical professionals, coaches. Health risk assessments are given based on available scientific evidence for that test. There are also some recommendations for physical fitness status available through the My SLOfit app– for example, explaining how one reaches or maintains a healthy level of physical fitness. The green zone means that the data is within this healthy, low-risk range. The yellow zone means that some improvements should be made to this test item. The red zone means significant improvement is necessary to prevent current (or future) health risks to the individual's overall health and wellbeing.

Since literature reviews failed to identify criterion-referenced standards for most tests, step 2 searched for dose-response relationships between a given fitness component and premature mortality across the life course. Two analyses on male youth found that 20% of people with the lowest cardiorespiratory fitness (32) and 10% of individuals with the lowest strength (33) were at the highest risk for premature death, with a gradual reduction in risk is seen across 3 next deciles. After the 4th decile, no clinically relevant additional benefits were observed. Similar results were found for the lowest 10% of youth with the lowest cardiorespiratory fitness (34) and strength in adolescence, and increased risk for all-cause disability 30 years after. Based on these, the 10th and 40th centile values were selected as cut-off points related to “unhealthy” and “less than optimal” fitness, respectively, for 6MWT, sit-ups, vertical jump, and all performance-related tests. Based on the evidence, three zones were specified: healthy fitness, needs improvement and health risk zone (Figure 1).

These procedures were repeated for the elderly, based on fitness components included in the SLOfit Senior fitness test battery. The search identified three studies which defined criterion-based standards for Senior fitness tests. We opted to primarily use a large, European-based study (35). For missing cut-off values, we relied largely on European normative data from Portugal (36). These normative data are available for all tests except the 2-min step test. Hence, for this test we used normative data from the U.S. (37). Finally, since Sardinha et al. (35) did not include “Chair Sit and Reach” or “Back Scratch” tests, we relied on criterion-referenced values in Chilean older adults reported by Merellano-Navarro et al. (38).

### Determination of normative references—population centiles

All pilot data collected from adults 19–64 years old were used to construct centile norms for each test item included in the SLOfit Adult test battery. Centile curves were obtained using the Lambda Mu Sigma (LMS) method separately for each gender following methods described elsewhere (39, 40). To note: pilot sampling was not nationally-representative, so statisticians used BMI data from a representative sample to calculate sample weights. In test items identical to those used in SLOfit Students (27), centiles curves for Adults were simply shifted downward or upwards to transition smoothly from childhood into adulthood. There is currently not enough data to confidently construct norms for SLOfit Seniors (65+ years).

### Individualized healthy feedback reports

After each measurement, participants enter their results into the application and receive immediate feedback on their physical fitness levels and health risks. Feedback can be interpreted as a relative change from different time-points, or by centile value compared to peers. BMI and fitness data in the My SLOfit application are presented according to health risk based on existing scientific evidence (outlined above, see Figure 1).

### My SLOfit infrastructure expansion

SLOfit system in supported by two web sites at URL *moj.slofit.org* and *www.slofit.org* (Slovenian and English), available to all web users offering program information for different user groups (children, parents, adults, teachers, physicians etc.). The My SLOfit website (URL: moj.slofit.org) is a secure web application running a DNN (formerly DotNetNuke) platform. All personal data are longitudinally saved in SQL database. Access to the data is initially granted only to the administrator of SLOfit testing providers (schools, sports clubs, health, or fitness centers etc.). If the participants of SLOfit fitness testing are registered users of MySLOfit, they can also access (their own) fitness data, complete questionnaires, and create reports, e.g., report of fitness testing (Figure 1). Registered users can share their own data with other people (e.g., physician, trainer) simply by entering their email address into the system which then sends an invitation for registration to that person. For all registered users the system is free of charge.

### Maximizing recruitment and retention

SLOfit Lifelong has a robust recruitment strategy in partnership with mainstream media, social media, and partner ambassadors who are well-known Slovenians (e.g., Olympic athletes, media personalities, comedians). In this way, SLOfit is building an online and real-world community. The free online My SLOfit app is customizable for use on different screen sizes (PCs, laptops, smartphones, tablets, etc.). The SLOfit website with the application is educational platform to increase physical and health literacy of children, adolescents, adults, and seniors. My SLOfit app allows individuals of all ages to (1) manage their chronic health conditions by regularly monitoring physical performance (2) encourage individuals to partake in regular exercise, (3) share their movement history with their doctor and (4) maintain a healthy lifestyle by following SLOfit expert advice based on their individual results.

### Citizen science engagement

SLOfit Lifelong encourages individuals to use data for their own exercise planning and to optimize the quality and quantity of their habitual movement behavior. Given the limited possibilities of communication between schools, physicians, municipalities, and parents it was increasingly important for us to establish holistic communication channels for the individual, their households, schools, and community environment. Since 2017/18, SLOfit registered users are already able to access information support through the SLOfit website (www.slofit.org) and free web application My SLOfit (moj.slofit.org), which provides free diagnostic tools, SLOfit reports expert advice in their native language (www.slofit.org/slofit-nasvet), and social media (Facebook, Instagram, YouTube) where citizens can engage directly with fitness, education and health professionals.

## Discussion

SLOfit Lifelong is a national physical fitness surveillance program that enables tracking fitness and movement behavior across the lifespan.

### Individual perspective

SLOfit Lifelong provides educational content *via* SLOfit reports and its social media presence (web magazine, Facebook, Instagram, YouTube). Each outlet provides unique tools and tips on how to increase one's health, fitness, and physical literacy. Health literacy is an important determinant of overall health, encouraging individuals to meet the demands of a modern society (41, 42) in a proactive manner. Those with greater health literacy have better health outcomes, healthcare services less frequently, with shorter hospitalization periods and increased medical adherence, motivation, and self-confidence (42, 43). The SLOfit App is specifically designed to assist individuals to understand their own health status and impart knowledge translation when one falls into a health risk zone.

### Clinical perspective

From a clinical perspective, SLOfit Lifelong conveys the message “from fitness diagnostics to health changes” by: (a) informing SLOfit participants about possible weaknesses in their fitness routine, and the importance of regular exercise (b) bond SLOfit participants to competent physical fitness activity providers (c) provide 2-way communication between patients and health care providers (d) enable health care professionals to monitor patient fitness to design appropriate, personalized physical activity programs.

### Public health perspective

SLOfit Lifelong provides a scientific backbone for creating/maintaining population-based policies related to improving physical fitness and activity. In future, causal relationships between children and adolescents' physical performance and future health outcomes can be inferred. Understanding to what extent physical fitness in childhood is a predictor of future disease will improve preventive interventions to increase physical fitness across the lifespan. With these program upgrades, SLOfit Lifelong will allow for detailed secular trends analyses in Slovenian subpopulations at the municipal, regional, and country level.

### Research perspective

From a research perspective, SLOfit Lifelong will develop a longitudinal cohort infrastructure using the Community-Based Participatory Research (CBPR) approach, one which involves direct participant engagement in which they can be affected by the research questions being addressed. Already, the SLOfit database includes about 8 million sets of measurements of over 1 million children. By enlarging SLOfit to include adulthood fitness surveillance, it is building a unique research platform for future studies to assess the predictive validity of health-related fitness.

### SLOfit Lifelong future vision

SLOfit Lifelong will continue as a participatory research platform by adding modules for sharing real world data such as smartwatches, fitness trackers, mobile phones, etc. It will be conceivable to link data from family members, enabling custom analyses, and providing evidence for at-risk, family-based interventions. The SLOfit team is closely following artificial intelligence technology that may allow the collection and analysis of large amounts of data on individual's movement behaviors (e.g., movement through geographical location, use of public transport), which could further improve the predictive models and enhance people's lifelong physical fitness.

## Conclusions

SLOfit Lifelong has developed unique physical fitness test batteries for adults which are physiologically comparable to those conducted in all Slovenian schools annually. By encasing the SLOfit Lifelong initiative within the existing SLOfit framework, web platform and infrastructure, researchers now have the capacity to store lifelong health and fitness data, including calculating health risk assessments, based on fitness data supplied by the citizen. Everyday people can follow their own fitness and health status, with expert feedback, to increase their physical literacy and fortify a sense of personal responsibility toward their own health outcomes.

## Data availability statement

The original contributions presented in the study are included in the article/supplementary material, further inquiries can be directed to the corresponding author/s.

## Ethics statement

The studies involving human participants were reviewed and approved by Slovenian National Medical Ethics Committee (ID: 0120-468/2021/3). The patients/participants provided their written informed consent to participate in this study.

## Author contributions

GJ: project administration, conceptualization, investigation, resources, writing- original draft, and writing- review and editing, writing- approving final submission. SM and VSe: writing- original draft, writing- review and editing, and writing- approving final submission. MS: conceptualization, writing- original draft, writing- review and editing, and writing- approving final submission. BL: conceptualization, investigation, resource, writing- original draft, writing- review and editing, and writing- approving final submission. MK: writing- review and editing and writing- approving final submission. TO: writing- original draft, investigation, resource, writing- review and editing, and writing- approving final submission. JK, KM, PG, VSt, and VH: investigation, resource, and writing- approving final submission. RB: investigation, resource, writing- review and editing, and writing- approving final submission. NM: investigation and writing- approving final submission. GS: conceptualization, investigation, resource, writing- review and editing, and writing- approving final submission. All authors contributed to the article and approved the submitted version.

## Funding

The SLOfit Lifelong initiative was co-funded by the Slovenian National Research Agency (J5-1797) and limited, non-specific funding from the research program group P5-0142 (Bio-psycho-social context of kinesiology).

## Conflict of interest

The authors declare that the research was conducted in the absence of any commercial or financial relationships that could be construed as a potential conflict of interest.

## Publisher's note

All claims expressed in this article are solely those of the authors and do not necessarily represent those of their affiliated organizations, or those of the publisher, the editors and the reviewers. Any product that may be evaluated in this article, or claim that may be made by its manufacturer, is not guaranteed or endorsed by the publisher.

## References

[1] Smith SC Jackson R Pearson TA Fuster V Yusuf S Faergeman O. Principles for national and regional guidelines on cardiovascular disease prevention: a scientific statement from the World Heart and Stroke Forum. Circulation. (2004) 109:3112–21. 10.1161/01.CIR.0000133427.35111.67 15226228

[2] Lee IM Shiroma EJ Lobelo F Puska P Blair SN Katzmarzyk PT. Effect of physical inactivity on major non-communicable diseases worldwide: an analysis of burden of disease and life expectancy. Lancet. (2012) 380:219–29. 10.1016/S0140-6736(12)61031-9 22818936 PMC3645500

[3] Ding D Lawson KD Kolbe-Alexander TL Finkelstein EA Katzmarzyk PT van Mechelen W. The economic burden of physical inactivity: a global analysis of major non-communicable diseases. Lancet. (2016) 388:1311–24. 10.1016/S0140-6736(16)30383-X 27475266

[4] McGill J McMahan CA Herderick EE Malcom GT Tracy RE Strong JP. Origin of atherosclerosis in childhood and adolescence. Am J Clin Nutr. (2000) 72(5 Suppl.):1307s–15s 10.1093/ajcn/72.5.1307s 11063473

[5] Strong JP Malcom GT Newman WP 3rd Oalmann MC. Early lesions of atherosclerosis in childhood and youth: natural history and risk factors. J Am Coll Nutr. (1992) 11(Suppl.):51S−4S. 10.1080/07315724.1992.10737984 1619200

[6] Raitakari OT Juonala M Kähönen M Taittonen L Laitinen T Mäki-Torkko N. Cardiovascular risk factors in childhood and carotid artery intima-media thickness in adulthood: the cardiovascular risk in young finns study. JAMA. (2003) 290:2277–83. 10.1001/jama.290.17.2277 14600186

[7] Clarke H. Academy approves physical fitness definition. Phys Fit Newsl. (1979) 25:1.

[8] Reilly JJ Kelly J. Long-term impact of overweight and obesity in childhood and adolescence on morbidity and premature mortality in adulthood: systematic review. Int J Obes. (2011) 35:891–8. 10.1038/ijo.2010.222 20975725

[9] Ruiz JR Castro-Piñero J Artero EG Ortega FB Sjöström M Suni J. Predictive validity of health-related fitness in youth: a systematic review. Br J Sports Med. (2009) 43:909–23. 10.1136/bjsm.2008.056499 19158130

[10] Glenister D. Exercise and mental health: a review. J R Soc Health. (1996) 116:7–13. 10.1177/146642409611600102 8683544

[11] Dwyer T Sallis J Blizzard L Lazarus R Dean K. Relation of academic performance to physical activity and fitness in children. Pediatr Exerc Sci. (2001) 13:225–37. 10.1123/pes.13.3.225

[12] Swallen KC Reither EN Haas SA Meier AM. Overweight, obesity, and health-related quality of life among adolescents: the National Longitudinal Study of Adolescent Health. Pediatrics. (2005) 115:340–7. 10.1542/peds.2004-0678 15687442

[13] Walston JD. Sarcopenia in older adults. Curr Opin Rheumatol. (2012) 24:623–7. 10.1097/BOR.0b013e328358d59b 22955023 PMC4066461

[14] Deschenes MR. Effects of aging on muscle fibre type and size. Sports Med. (2004) 34:809–24. 10.2165/00007256-200434120-00002 15462613

[15] Mitchell WK Williams J Atherton P Larvin M Lund J Narici M. Sarcopenia, dynapenia, and the impact of advancing age on human skeletal muscle size and strength; a quantitative review. Front Physiol. (2012) 3:260. 10.3389/fphys.2012.00260 22934016 PMC3429036

[16] Delmonico MJ Harris TB Visser M Park SW Conroy MB Velasquez-Mieyer P. Longitudinal study of muscle strength, quality, and adipose tissue infiltration. Am J Clin Nutr. (2009) 90:1579–85. 10.3945/ajcn.2009.28047 19864405 PMC2777469

[17] Hodgson JL Buskirk ER. Physical fitness and age, with emphasis on cardiovascular function in the elderly. J Am Geriatr Soc. (1977) 25:385–92. 10.1111/j.1532-5415.1977.tb00671.x 330606

[18] Stathokostas L McDonald MW Little RMD Paterson DH. Flexibility of older adults aged 55-86 years and the influence of physical activity. J Aging Res. (2013) 2013:743843. 10.1155/2013/743843 23862064 PMC3703899

[19] Balogun JA Akindele KA Nihinlola JO Marzouk DK. Age-related changes in balance performance. Disabil Rehabil. (1994) 16:58–62. 10.3109/09638289409166013 8043885

[20] Spirduso WW Cronin DL. Exercise dose-response effects on quality of life and independent living in older adults. Med Sci Sports Exerc. (2001) 33(6 Suppl.):S598–S608. 10.1097/00005768-200106001-00028 11427784

[21] Rantanen T Guralnik JM Foley D Masaki K Leveille S Curb JD. Midlife hand grip strength as a predictor of old age disability. JAMA. (1999) 281:558–60. 10.1001/jama.281.6.558 10022113

[22] Hunt A. Musculoskeletal fitness: the keystone in overall well-being and injury prevention. Clin Orthop Relat Res. (2003) 409:96–105. 10.1097/01.blo.0000057787.10364.4e 12671491

[23] Katzmarzyk PT Craig CL. Musculoskeletal fitness and risk of mortality. Med Sci Sports Exerc. (2002) 34:740–4. 10.1097/00005768-200205000-00002 11984288

[24] Beaudart C Zaaria M Pasleau F Reginster JY Bruyère O. Health outcomes of sarcopenia: a systematic review and meta-analysis. PLoS ONE. (2017) 12:e0169548. 10.1371/journal.pone.0169548 28095426 PMC5240970

[25] Ortega FB Ruiz JR Castillo MJ Sjöström M. Physical fitness in childhood and adolescence: a powerful marker of health. Int J Obes. (2008) 32:1–11. 10.1038/sj.ijo.0803774 18043605

[26] Ross R Blair SN Arena R Church TS Després JP Franklin BA. Importance of assessing cardiorespiratory fitness in clinical practice: a case for fitness as a clinical vital sign: a scientific statement from the American Heart Association. Circulation. (2016) 134:e653–99. 10.1161/CIR.0000000000000461 27881567

[27] Jurak G Leskošek B Kovač M Sorić M Kramaršič J Sember V. SLOfit surveillance system of somatic and motor development of children and adolescents: upgrading the Slovenian Sports Educational Chart. Auc Kinanthropol. (2020) 56:28–40. 10.14712/23366052.2020.4

[28] Jurak G Kovač M Sember V Starc G. 30 years of SLOfit: its legacy and perspective. Turkish J Sport Med. (2019) 54(Supp1.):23–7. 10.5152/tjsm.2019.148

[29] Kovač M Jurak G Starc G Leskošek B Strel J. Športnovzgojni karton : diagnostika in ovrednotenje telesnega in gibalnega razvoja otrok in mladine v Sloveniji. Univerza v Ljubljani, Fakulteta za šport (2011). Available online at: https://www.fsp.uni-lj.si/mma/Porocilo_SVK_06-07.pdf/20150920202452/?m=1442773492 (accessed August 23, 2022).

[30] Riebe D editor. ACSM's Guidelines for Exercise Testing and Prescription. 10th ed. Philadelphia, PA: Wolters Kluwer (2018).

[31] Rikli RE Jones CJ. Senior Fitness Test Manual. 2nd ed. Champaign, IL: Human Kinetics (2013).

[32] Högström G Nordström A Nordström P. Aerobic fitness in late adolescence and the risk of early death: a prospective cohort study of 13 million Swedish men. Int J Epidemiol. (2016) 45:1159–68. 10.1093/ije/dyv321 26686843

[33] Ortega FB Silventoinen K Tynelius P Rasmussen F. Muscular strength in male adolescents and premature death: cohort study of one million participants. BMJ. (2012) 345:e7279. 10.1136/bmj.e7279 23169869 PMC3502746

[34] Henriksson P Henriksson H Tynelius P Berglind D Löf M Lee IM. Fitness and body mass index during adolescence and disability later in life: a cohort study. Ann Intern Med. (2019) 170:230–9. 10.7326/M18-1861 30743265 PMC6814012

[35] Sardinha LB Santos DA Marques EA Mota J. Criterion-referenced fitness standards for predicting physical independence into later life. Exp Gerontol. (2015) 61:142–6. 10.1016/j.exger.2014.12.012 25528601

[36] Marques EA Baptista F Santos R Vale S Santos DA Silva AM. Normative functional fitness standards and trends of Portuguese older adults: cross-cultural comparisons. J Aging Phys Act. (2014) 22:126–37. 10.1123/japa.2012-0203 23538513

[37] Rikli RE Jones CJ. Functional fitness normative scores for community-residing older adults, ages 60-94. J Aging Phys Act. (1999) 7:162–81. 10.1123/japa.7.2.162

[38] Merellano-Navarro E Collado-Mateo D García-Rubio J Gusi N Olivares PR. Criterion-referenced fitness standards associated with maintaining functional capacity in chilean older adults. Rejuvenation Res. (2017) 20:484–91. 10.1089/rej.2016.1913 28514190

[39] Radulović A Jurak G Leskošek B Starc G Blagus R. Secular trends in physical fitness of Slovenian boys and girls aged 7 to 15 years from 1989 to 2019: a population-based study. Sci Rep. (2022) 12:10495. 10.1038/s41598-022-14813-7 35729360 PMC9213534

[40] Blagus R Jurak G Starc G Leskošek B. Centile reference curves of the SLOfit physical fitness tests for school-aged children and adolescents. J Strength Cond Res. (2022). 10.1519/JSC.0000000000004265. [Epub ahead of print]. 35900799 PMC9872862

[41] Fleary SA Joseph P Pappagianopoulos JE. Adolescent health literacy and health behaviors: a systematic review. J Adolesc. (2018) 62:116–27. 10.1016/j.adolescence.2017.11.010 29179126

[42] Sørensen K Van Den Broucke S Fullam J Doyle G Pelikan J Slonska Z. Health literacy and public health: a systematic review and integration of definitions and models. BMC Public Health. (2012) 12:80. 10.1186/1471-2458-12-80 22276600 PMC3292515

[43] Parker R. Health literacy: a challenge for American patients and their health care providers. Health Promot Int. (2000) 15:277–83. 10.1093/heapro/15.4.277

